# Evaluating the long-term efficacy of umbilical cord-derived mesenchymal stem cell therapy in retinitis pigmentosa: findings from a 1-to-4-year follow-up

**DOI:** 10.1093/stcltm/szaf034

**Published:** 2025-09-04

**Authors:** Ayse Oner, Neslihan Sinim Kahraman, Ali Unal

**Affiliations:** Department of Ophthalmology, Acibadem Taksim and Kayseri Hospital, Istanbul 34718, Turkiye; Department of Ophthalmology, Acibadem University, Istanbul 34718, Turkiye; Professor in Hematology Department and Bone Marrow Transplantation Center at Erciyes University, Kayseri 38039, Turkiye

**Keywords:** cell-mediated therapy, retinitis pigmentosa, suprachoroidal, umbilical cord-derived mesenchymal stem cells, visual function

## Abstract

**Background:**

The objective of this study was to assess the long-term effectiveness and safety of implanting mesenchymal stem cells derived from umbilical cord tissue (UC-MSC) in patients diagnosed with retinitis pigmentosa (RP).

**Methods:**

In this single-center study with a retrospective design, 669 eyes received suprachoroidal implantation of 5 million UC-MSCs. Postoperative assessments were conducted on the first day, third month, and every 6 months thereafter. At each visit, evaluations included best-corrected visual acuity (BCVA), anterior segment and fundus examinations, fundus photography, optical coherence tomography, and visual field (VF) tests. Multifocal electroretinography (mfERG) and full-field stimulus threshold (FST) testing were performed at baseline and every 6 months post-therapy. Procedure-related ocular and systemic complications were methodically documented.

**Results:**

A total of 669 eyes from 429 patients underwent surgical intervention. Bilateral procedures were performed in 240 patients, while 189 patients received surgery in only 1 eye. All 669 eyes completed the 12-month follow-up, while 265 eyes completed 2 years, 128 eyes completed 3 years, and 19 eyes completed 4 years of follow-up. No notable ocular or systemic complications were reported during the study duration. Statistically significant improvements in BCVA, VF, and mfERG central rings amplitude measurements were observed over time. FST testing revealed significant improvements in visual sensitivity in 27 patients.

**Conclusions:**

This investigation confirms the long-term benefits and safety profile of suprachoroidal UC-MSC therapy in cases of RP, demonstrating significant improvements in BCVA, VF, mfERG, and FST test outcomes. The data support the feasibility and potential of cell-based therapies as a promising and effective strategy for managing degenerative retinal diseases.

## Graphical Abstract

**Graphical Abstract:**
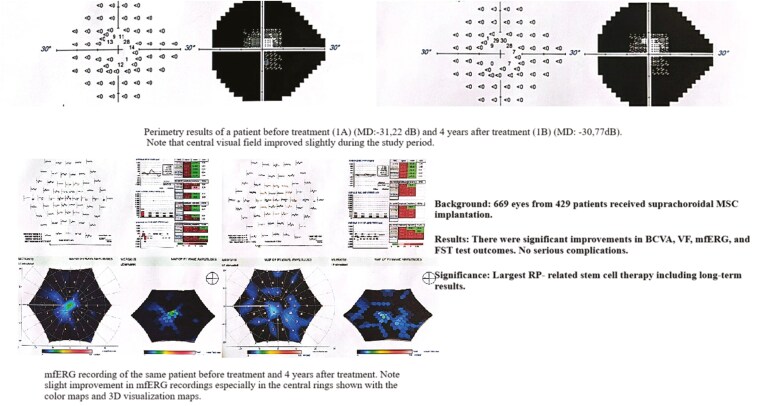

**Significance Statement**

In this study, the authors carried out suprachoroidal implantation of umbilical cord-derived mesenchymal stem cells (UC-MSCs) and reported no serious ocular adverse events in a large clinical case series. To the best of our knowledge, this constitutes the largest stem cell study to date, encompassing 669 eyes affected by retinitis pigmentosa (RP) and including a follow-up period of up to 4 years. The findings demonstrate that UC-MSC therapy significantly slows and stabilizes the progression of RP over the long term, highlighting its potential as a safe and effective treatment option for this degenerative retinal disease.

## Introduction

Retinitis pigmentosa (RP) refers to a heterogeneous group of genetic retinal diseases marked by the gradual degeneration of photoreceptor cells. The condition is primarily caused by mutations that impair the functionality and viability of retinal cells. RP typically begins with the degeneration of rod cells, leading to night blindness and peripheral vision loss (tunnel vision), and subsequently affects cone cells, resulting in complete visual impairment in advanced stages. Patients with RP may also experience challenges in color perception and sensitivity to light.^1,2^

Although a definitive cure for RP has yet to be established, multiple therapeutic strategies are under investigation to mitigate symptoms and decelerate disease progression. These include gene therapy, stem cell therapies, and supportive interventions such as genetic counseling, low-vision aids, and orientation and mobility training to enhance patients’ independence. Genetic counseling is particularly valuable in helping individuals understand the hereditary aspects of RP and make informed family planning decisions. Gene therapy has shown significant promise, with Voretigene neparvovec-rzyl (LUXTURNA, Spark Therapeutics, Philadelphia, PA). On December 19, 2017, FDA approval of this drug was granted for the treatment of individuals diagnosed with retinal dystrophy associated with confirmed biallelic RPE65 mutations. Numerous ongoing clinical trials are investigating therapies for other genetic mutations linked to RP. However, these gene-specific approaches leave a significant proportion of RP cases without targeted treatment options.^3-6^

In recent years, the landscape of regenerative medicine, particularly stem cell therapies, has emerged as a promising option for retinal restoration. Among the various stem cell types under investigation, mesenchymal stem cells (MSCs) have attracted significant attention for their therapeutic potential. MSCs, derived from various sources, including umbilical cord tissue, bone marrow, and adipose tissue, exhibit trophic and immunomodulatory effects that may mitigate the degenerative processes in the retina.^7-9^

Experimental studies have emphasized the safety and efficacy of MSC transplantation in preclinical models of RP, demonstrating improvements in visual function and the preservation of retinal structure.^10,11^While the initial clinical findings regarding MSC therapies for RP are promising,^12-17^ the translation of these applications to long-term clinical success is a critical point in the future.

This study aimed to assess the long-term safety and therapeutic effect of umbilical cord-derived mesenchymal stem cell (UC-MSC) implantation in patients with RP. By assessing long-term outcomes, this research attempts to contribute valuable insights to the growing knowledge in regenerative ophthalmology and to prepare the way for future advancements in the treatment of retinal degenerative disorders.

## Methods

### Study design

This retrospective clinical study was conducted to assess the long-term safety and efficacy of suprachoroidal implantation of UC-MSCs in patients diagnosed with RP. The study included patients treated with stem cell therapy at the ophthalmology department of our hospital between March 1, 2019, and December 1, 2023. The study was conducted in compliance with the principles outlined in the Declaration of Helsinki, following approval by the University Ethics Committee. (Approval No. 2017/480, October 13, 2017) and authorization from the Ministry of Health’s Review Board for Stem Cell Applications (Registration No. 56733164/203), as per national regulations. Written informed consent was obtained from all participants before their enrollment in the study.

### Patient selection

Following a thorough examination of the patients’ complete medical histories, their eligibility was assessed based on the inclusion and exclusion criteria. The inclusion criteria for the study were as follows: (1) age of 18 years or older,^2^ A clinical diagnosis of RP confirmed through ophthalmological tests,^3^ having best-corrected visual acuity (BCVA) below 20/50, and^4^ exhibiting varying degrees of visual field (VF) loss. The exclusion criteria involved: (1) undergoing previous ocular surgeries other than cataract extraction,^2^ having ocular media opacities that could compromise ocular imaging or impact multifocal electroretinography (mfERG) or VF assessment,^3^ experiencing concurrent ocular conditions such as retinal pathology, uveitis, strabismus, or nystagmus,^4^ suffering from systemic diseases that might influence the study results, and (5) maintaining a smoking habit.

### Variables and outcomes

Upon documenting demographic factors, each patient underwent an extensive ophthalmic assessment, including measurements of BCVA, intraocular pressure, slit-lamp biomicroscopy for anterior segment evaluation, color fundus photography, optical coherence tomography (OCT), VF, mfERG, and full-field stimulus threshold (FST) tests.

The primary outcomes of our study were to evaluate BCVA, VF, mfERG, and FST testing before and after treatment. VF examinations were conducted using the Humphrey VF analyzer device (Carl Zeiss Meditec AG, Germany) with program 24-2 employed for testing each eye. BCVA was recorded using a Snellen chart at a 3-m distance and presented as Snellen Lines. MfERG recordings were obtained with the mfERG Vision monitor (Metrovision, France), following the guidelines of the International Society for Clinical Electrophysiology of Vision.^18^ During mfERG evaluations, a matrix comprising 61 hexagons, each corresponding to an individual mfERG response, was generated. These hexagons were arranged into 5 concentric rings (<2°, 2-5°, 5-10°, 10-15°, and >15°) centered on the fovea. The mean amplitude and implicit time of the first positive wave (P1) were recorded for each ring.

FST testing is a psychophysical assessment used to measure the sensitivity of the retina to light stimuli, particularly in patients with severe visual impairment, such as those with RP. The test evaluates the minimum light intensity that can elicit a visual response, serving as a functional marker of retinal activity. For this study, the MonVision (Metrovision, France) device was utilized to perform FST testing. This device presents diffuse light stimuli of varying wavelengths, including white, blue, and red, over the entire VF to evaluate rod- and cone-mediated visual responses. The testing procedure is conducted in a completely darkened environment to ensure maximal pupil dilation and adaptation to darkness for at least 40 minutes. The patient is instructed to indicate the perception of the light stimulus by pressing a button. The test determines the lowest stimulus intensity that the patient can detect and records thresholds in decibels (dB), with higher dB values indicating better retinal sensitivity. FST testing has been available in the department for the past year.

An additional primary focus of the study was to report adverse events associated with stem cell implantation and the surgical procedure. Adverse events were defined as the occurrence of any ocular or systemic complications.

### Stem cell preparation process

The umbilical cord underwent a disinfection process and was then meticulously sectioned into pieces measuring 1-2 mm² each. The tissue fragments were subsequently transferred into 75 cm² culture flasks containing Dulbecco’s Modified Eagle’s Medium-low glucose (DMEM-LG) supplemented with 10% human serum and 1% penicillin/streptomycin. Cultures were incubated at 37°C in a humidified atmosphere with 5% CO₂. Every 3 days, the culture medium was refreshed, and the process continued until reaching 70% confluency. Cells at the third passage, expanded through culture, underwent scrutiny for surface protein expression using flow cytometry. The UC-MSCs exhibited positive expression for CD-73, CD-90, and CD-105, while displaying negativity for CD-34, CD-45, and HLA-DR. Prior to release, thorough testing confirmed the absence of bacterial or fungal contamination in the cells. Cell viability, evaluated using the trypan blue dye exclusion assay, exceeded 90.0% ± 0.5% prior to transplantation. A suspension containing 5 × 10⁶ cells in a 0.7 mL isotonic solution supplemented with 1% human serum albumin was carefully aliquoted into vials under temperature-controlled conditions. The product was utilized within 12 hours of preparation and administered within the subsequent 24 hours.

### Surgical interventions

A total of 669 eyes from 429 patients underwent surgical intervention. Bilateral procedures were performed in 240 patients, while 189 patients received surgery in only 1 eye. In cases with bilateral surgery, each eye was operated on at separate time points, with a minimum interval of 3 months between the 2 procedures. The eye with poorer visual acuity was prioritized for the initial surgery. Similarly, in patients who underwent unilateral surgery, the eye with lower baseline visual acuity was selected for the procedure.

All of the surgical procedures were conducted by an experienced surgeon (AO) and performed under local anesthesia. The approach performed in these surgeries adhered to the Limoli retinal restoration technique, as detailed by Limoli et al.^13^ In the current study, we adhered to the same Limoli retinal restoration technique, with a modification in the stem cell source and quantity.

The details of the surgery are as follows: The globe was deviated to the supero-nasal quadrant, and the conjunctiva was dissected at the infero-temporal quadrant at 8 mm from the limbus. A deep scleral flap of about 5 × 5 mm was opened by radial hinge at the infero-temporal quadrant. The sclerectomy was deep enough to allow viewing the color of the choroid. A flap of the orbital fat was extracted from a gap above the inferior oblique muscle. This tissue was laid on the scleral bed and sutured with 6/0 Vicryl at the proximal edge. The scleral flap was then sutured above the fat pedicle. The remaining space between the autologous fat graft, choroid, and scleral flaps was filled with 0.7 cc of 5 × 10^6^ UCMSCs using a 25-gauge cannula. The conjunctiva was sutured with 8/0 Vicryl.

### Postoperative follow-up period

Follow-up after surgery involved a 1-day hospital stay. Patients are recommended to apply topical antibiotic and steroid eye drops 4 times daily for a month following the procedure. Ophthalmic assessments, including BCVA, examinations of the anterior and posterior segments, color fundus photography, and OCT scans, were conducted preoperatively on the first day, first month, third month, and every sixth month postoperatively. VF tests were performed at the first, third, and then every 6 months. mfERG and FST tests were conducted every 6 months after the surgery. Throughout the study period, all patients were monitored for any adverse events related to the surgical procedures.

### Statistical analysis

Statistical analyses were performed using the SPSS software package, version 20 (IBM Corp., Armonk, NY). Descriptive statistics for non-normally distributed numerical variables are presented as median and interquartile range, while categorical variables are reported as frequencies and percentages. The Shapiro–Wilk and Kolmogorov–Smirnov tests were applied to assess the distribution of numerical data. Comparisons of VF and BCVA measurements were conducted using the Related-Samples Friedman’s Two-Way Analysis of Variance by Ranks test, followed by post hoc pairwise comparisons using the Wilcoxon signed-rank test with Bonferroni correction. MfERG and FST measurements were analyzed using the Wilcoxon signed-rank test. A *P*-value of <.05 was considered statistically significant.

## Results

The mean age of 429 patients was 35.6 years (range: 18-61 years). Follow-up results were available for 669 operated eyes at 1 year, 265 eyes at 2 years, 128 eyes at 3 years, and 19 eyes at 4 years. No systemic or serious ocular side effects were observed in any of the cases. The most common ocular side effects were surgery-related conjunctival hyperemia and edema (67%) and increased light sensitivity (18%). BCVA results are presented in Table 1, VF-MD values in Table 2, mfERG test results in Table 3, and FST test results in Table 4. No additional retinal pathologies were detected on OCT during the follow-up period.

**Table 1.** Comparison of mean BCVA in Snellen lines (Decimal and Metric).

Visit time	*n*	Mean (decimal-metric)	Median (Min-Max) (Decimal)	Median (Min-Max) (metric)	*P*-value
Baseline	669	0,168 (20/119)^a^	0.130 (0.003-0.800)^a^	20/154 (20/6667-20/25)^a^	
1 month	669	0.200 (20/100)^b^	0.170 (0.003-0.900)^b^	20/118 (20/6667-20/22)^b^	<.001
6 month	669	0.219 (20/91)^b^	0.170 (0.010-1.000)^b^	20/118 (20/2000.20/20 or better)^b^	<.001
1 year	669	0.220 (20/91)^b^	0.150 (0.005-1.000)^b^	20/133 (20/4000-20/20 or better)^b^	<.05
2 year	265	0.303 (20/66)^b^	0.140 (0.010-1.000)^b^	20/143 (20/2000-20/20 or better)^b^	<.05
3 year	128	0.250 (20/80)^b^	0.150 (0.005-1.000)^b^	20/133 (20/4000-20/20 or better)^b^	<.05
4 year	19	0.172 (20/116)^b^	0.140 (0.010-1.000)^b^	20/143 (20/2000-20/20 or better)^b^	<.05

a,b: The difference between measurements with a and b is significant.

**Table 2.** Comparison of mean VF-MD values in decibels.

Visit time	*n*	Mean ± SD	Median (Min/Max)
Baseline	669	,28 560 ± −5623	−29 450 (−21 810/−34 590)^a^
1 month	669	−28 442 ± −5262	−27 870 (−21 810/−34 590)^b^
6 month	669	−28 121 ± −5114	−26 685 (−19 260/−34 590)^b^
1 year	669	−27 919 ± −5104	−26 520 (−19 130/−32 975)^b^
2 year	265	−27 202 ± −5209	−27 720 (−20 680/−34 650)^b^
3 year	128	−27 682 ± −5109	−26 120 (−20 130/−32 975)^b^
4 year	19	−27 692 ± −5269	−26 105 (−20 160/−32 975)^b^

SD, standard deviation.

a,b: The difference between measurements with a and b is significant. (*P* < .05).

**Table 3.** Comparison of mean P1 amplitudes (µV) of mf ERG rings.

Visit time	*n*	Ring 1 <2° P1 ampl	Ring 2 2-5° P1 ampl	Ring 3 5-10° P1ampl	Ring 4 10-15° P1 ampl	Ring 5 >15° P1 ampl
Baseline	648	330.7 ± 197.4^a^	196.7 ± 138.7^a^	115.3 ± 84.8	75.9 ± 51.0	52.9 ± 38.9
6 month	648	361.8 ± 217.6^b^	239.3 ± 151.1^b^	111.8 ± 98.3	73.1 ± 49.7	49.7 ± 28.6
1 year	642	355.4 ± 192.3^b^	241.6 ± 165.2^b^	109.6 ± 88.2	70.6 ± 45.4	45.2 ± 32.5
2 year	257	351.6 ± 201.4^b^	237.3 ± 123.6^b^	113.3 ± 78.6	69.4 ± 37.8	50.2 ± 35.6
3 year	128	348.5 ± 196.2^b^	228.3 ± 138.7^b^	116.9 ± 68.0	72.2 ± 39.5	46.6 ± 31.7
4 year	19	349.9 ± 187.2^b^	219.3 ± 142.3^b^	109.8 ± 67.0	62.8 ± 59.2	47.6 ± 37.8

a,b: The difference between measurements with a and b is significant. (*P* < .05).

**Table 4.** Comparison of FST values in decibels.

Visit time	*n*	White FST	Red FST	Blue FST
Baseline, dB	27	43.9 ± 13.9^a^	40.5 ± 12.5^a^	51.2 ± 16.2^a^
6 month, dB	27	50.3 ± 16.8^b^	46.5 ± 11.6^b^	59.3 ± 19.4^b^
1 year, dB	27	53.6 ± 14.7^b^	49.8 ± 16.7^b^	62.3 ± 17.8^b^

dB, Decibel.

a,b: The difference between measurements with a and b is significant. (*P* < .05).

### Functional assessment parameters

#### Mean BCVA

A statistically significant improvement was observed in BCVA during the first 2 years after treatment. Baseline BCVA was 0.168 Snellen lines. It improved to 0.220 in the first year and further increased to 0.303 in the second year. However, it declined to 0.250 in the third year and 0.172 in the fourth year, yet it remained higher than the baseline value (Table 1; *P* < .05).

#### Mean VF-MD

A statistically significant increase in MD was detected during the first 2 years after treatment. The baseline mean deviation (MD) was −28.560 decibels (dB). It improved to −27.919 in the first year and −27.202 in the second year. Although there was some deterioration in the third and fourth years, the results remained better than the baseline, with values of −27.682 in the third year and −27.692 in the fourth year. (Table 2; *P* < .05).

#### mfERG

Significant improvements were observed in the amplitudes of the P1 waves in the central rings (<2° and 2-5°) at 2 years postoperatively. In contrast, the amplitudes of P1 waves in the peripheral rings (5-10°, 10-15°, and >15°) showed slight decreases compared to preoperative values, although these differences were not statistically significant (Table 3; *P* < .05).

#### Full-field stimulus threshold

FST test results demonstrated statistically significant improvements in light sensitivity thresholds for white, blue, and red light during the 1-year follow-up period (Table 4; *P* < .05). Baseline FST results were 43.9 dB for white, 40.5 dB for red, and 51.2 dB for blue stimuli. After 1 year of therapy, these values improved to 53.6 dB, 49.8 dB, and 62.3 dB, respectively.

#### OCT findings

OCT scans of all treated eyes showed no signs of additional fluid accumulation, edema, or morphological changes during follow-up. Mean central macular thickness measurements did not change significantly after treatment. Two eyes developed retinal detachment 2 years after stem cell therapy, which were successfully treated without complications. Both cases involved highly myopic eyes, and one of them was pseudophakic. Regarding the timing and condition of the eyes with retinal detachment, the cases were considered to be coincidental.

**Figure 1.**
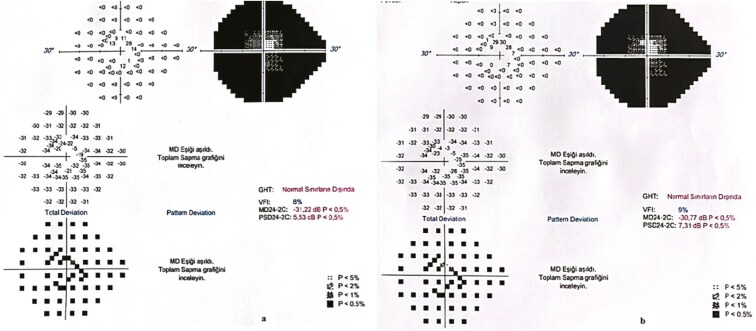
Perimetry results of a patient before treatment (A) (MD:−31.22 dB) and 4 years after treatment (B) (MD: −30.77dB). Note that the central visual field improved slightly during the study period.

**Figure 2.**
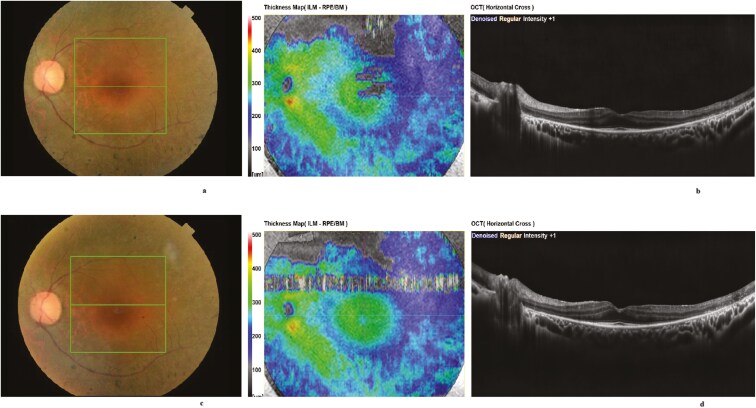
Fundus photographs (A) and OCT (B) of the same patient taken before treatment and 4 years after treatment (Figure 2C and D). Notably, the central ellipsoid zone band appears preserved throughout the follow-up period.

**Figure 3.**
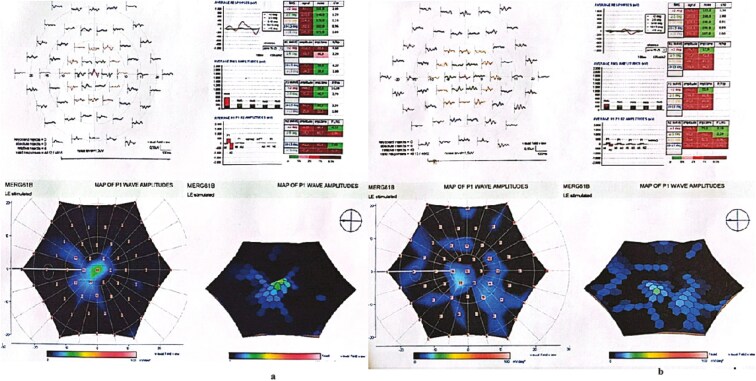
mfERG recording of the same patient before treatment (A) and 4 years after treatment (B). Note slight improvement in mfERG recordings, especially in the central rings shown with the color maps and 3D visualization maps.

**Figure 4.**
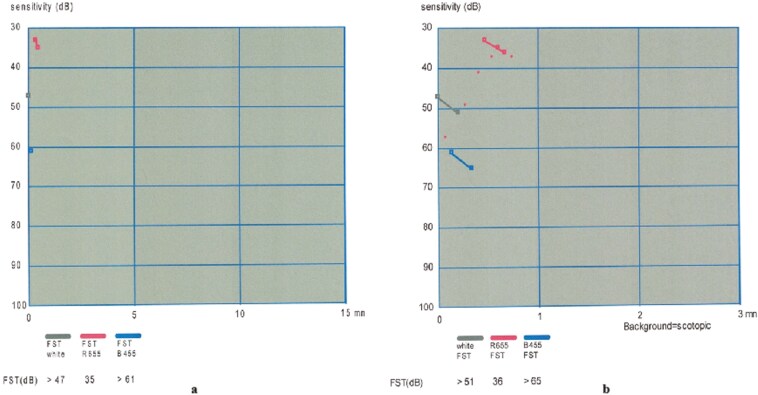
FST recording of another patient before treatment (A) (White FST: 47 dB Red FST 35 dB and Blue FST 61 dB) and 12 months after treatment (B) (White FST: 51 dB Red FST 36 dB and Blue FST 65 dB). Note the improvements in the recordings of white, red, and blue FST.

Representative examples of VF, OCT, mfERG of 1 patient, and FST from another patient are presented in Figures 1-4.

#### Adverse events

No serious ocular and systemic complications were reported during the follow-up period.

## Discussion

Visual impairment significantly complicates daily activities, and the progressive loss of vision associated with retinal disorders severely diminishes quality of life.^19^ Although no definitive cure currently exists for degenerative retinal diseases, extensive research efforts are focused on exploring potential treatments, including gene therapy and stem cell-based approaches. Among these, stem cell-based therapies have attracted considerable attention due to their potential to reverse cellular damage and enhance the function of remaining viable retinal cells. Recent studies on stem cell applications in ophthalmology, particularly for hereditary and age-related retinal disorders as well as optic neuropathies, have yielded promising outcomes.^12-17^

Multiple mechanisms have been hypothesized to explain photoreceptor degeneration in RP, including deficiencies in trophic factors, increased oxidative stress, activation of pro-inflammatory microglia, and nutrient deprivation.^1^ These combined mechanisms support numerous preclinical and clinical studies demonstrating that UC-MSCs can reactivate dormant photoreceptors, enhance retinal functions, prevent photoreceptor apoptosis, and slow the progression of RP and other retinal degenerative disorders. These integrated mechanisms provide the biological basis for extensive preclinical and clinical evidence demonstrating that UC-MSCs facilitate the reactivation of dormant photoreceptors, augment retinal functional capacity, inhibit photoreceptor apoptosis, and slow the progression of RP and other retinal degenerative conditions.^11,12,14-17,20^

Limoli outlined the potential therapeutic mechanisms of the stem cell approach as follows^13^:

Hemorheological Activity: Restoration of retinal blood flow.

Anti-Oxidative Activity: Prevention of oxygen-induced photoreceptor degeneration in the retina.

Anti-Inflammatory Activity: Modulation of microglial activation, which occurs concurrently with or precedes peak apoptotic photoreceptor death in RP.

Anti-Apoptotic Activity: Regulation of cytokines that influence cell survival or apoptosis.

Cytoprotective Activity of Growth Factors (GFs): Neuroprotective support through the regulation of photoreceptor metabolic activity.

Significant advancements in stem cell therapies for RP have been achieved in recent years. However, intravitreal and subretinal injection methods for MSC implantation have raised safety concerns due to reported complications.^21-23^ A recent study involving 11 severe RP patients who underwent subretinal application of adipose-derived MSCs reported ocular complications in 6 cases. One patient developed a choroidal neovascular membrane requiring intravitreal anti-VEGF treatment, while 6 participants experienced epiretinal membrane formation with peripheral tractional retinal detachment, necessitating a second vitrectomy. These adverse events were attributed to factors such as vitreal reflux, unintended preretinal MSC injection, aberrant MSC proliferation, or pre-existing vitreous abnormalities in advanced RP cases that hindered proper vitreous removal. Despite these issues, 4 subjects experienced vision improvement during the first year of follow-up. The authors cautioned that subretinal MSC implantation carries a risk of adverse effects, highlighting the need for careful patient selection and monitoring.^23^

Given the complications associated with intravitreal and subretinal delivery methods, alternative extraocular approaches, including suprachoroidal, subtenon, or peribulbar routes, have been recommended as safer options. These methods have demonstrated favorable safety profiles in recent studies.^12-16,20,24–26^

In a recent phase 3 clinical study, Özmert et al. explored the use of Wharton’s jelly-derived MSCs (WJ-MSCs) for RP. The study involved 32 RP patients who received WJ-MSC implantation into the subtenon space.^12^ At the 6-month follow-up, mean BCVA improved significantly from 70.5 to 80.6 letters. Concurrent enhancements were detected in VF-MD values and outer retinal thickness. mfERG analyses demonstrated significant increases in P1 wave amplitudes and implicit times of central retinal zones (rings 1-3), accompanied by improved full-field ERG cone responses. The authors proposed that while peripheral photoreceptors had largely undergone apoptosis, central photoreceptors remained dormant and viable for functional recovery. Importantly, no ocular or systemic side effects related to the WJ-MSC implantation were reported during the study’s follow-up period.^14^ The 12-month outcomes of the same study^15^ involved 34 eyes from 32 patients with different RP genotypes. Genetic analyses were performed using an RP panel sequencing method targeting 90 relevant genes. At the 1-year follow-up, patients with autosomal dominant and autosomal recessive RP showed significant increases in BCVA, as well as in ERG amplitudes and implicit times. However, no significant improvements were detected in subjects with X-linked RP. Notably, there were no ocular or systemic side effects related to the surgical procedure or the WJ-MSCs during the 12-month follow-up period. Long-term follow-up results, extending to 36 months^20^ suggested that the combined use of WJ-MSCs and high-frequency repetitive electromagnetic stimulation (Magnovision, Bioretina Biotechnology) was the most effective strategy for slowing disease progression and maintaining retinal function, compared to using either WJ-MSCs or Magnovision alone. Treatment with WJ-MSCs alone was more effective than Magnovision alone in decelerating disease progression. Most patients reported subjective improvements in daily activities during follow-up assessments. However, these subjective improvements require formal validation through standardized tools, such as the Quality of Life Index.

In a recent prospective clinical case series, Ozkan et al. evaluated the effects of suprachoroidal application of MSCs in the form of spheroids as a therapeutic approach for RP patients with relatively preserved visual acuity.^26^ The study evaluated 15 eyes from 15 participants. At baseline, the median BCVA was recorded as 1.30 logMAR. Postoperatively, the median BCVA improved to 1.00 at 1 month, 0.80 at 3 months, and remained stable at 0.80 at 6 months. The improvements observed at the 3 and 6-month follow-up visits were statistically significant compared to baseline values. In addition to BCVA improvements, significant enhancements were observed in the median MD values of both the 30-2 and 10-2 VF tests. Furthermore, mfERG results demonstrated significant increases in the amplitudes of P1 waves in the central 3 rings, along with increases in the implicit times of P1 waves in the 10°-15° ring by the sixth postoperative month.

The authors concluded that suprachoroidal application of MSCs in the form of spheroids demonstrates a regenerative effect in RP patients with relatively preserved visual acuity, leading to statistically significant increases in BCVA, VF performance, and mfERG results over a 6-month postoperative period. Although the spheroid form of MSCs differs from other approaches, the results provide clear evidence that MSC therapy via suprachoroidal implantation is both safe and effective in promoting retinal function in RP patients with preserved visual acuity.

Recent progress in gene and stem cell treatments has necessitated the enrollment of patients with low vision in clinical trials to assess therapeutic outcomes effectively. Clinical studies, particularly those involving low-vision patients, have highlighted the critical importance of the FST test as an evaluation tool.

Previous gene therapy studies have indicated that in patients with severe visual impairment, reliable responses could not be achieved with conventional electrophysiological tests. It has become evident that, among available modalities, only the FST test is suitable for evaluating treatment outcomes in individuals with very low vision.^27,28^

In a recent clinical study involving RP patients in the intermediate and late stages of the disease, all participants were able to reliably perform FST tests. The FST results were negatively correlated with age and disease duration, while positive correlations were observed with BCVA, VF MD values, central macular thickness, and ellipsoid zone band width.^29^

According to the literature to date, this is the first study to include FST results following stem cell therapy. Among the 27 eyes treated over the past year, all were able to perform the FST test. Significant improvements in mean white, red, and blue FST test values were detected at the 1-year results.

In this current investigation, the authors conducted UC-MSC implantation into the suprachoroidal space and observed no serious ocular complications in this extensive clinical study. To the best of our understanding, this constitutes the most extensive stem cell study to date, including 669 eyes with RP, and boasting the lengthiest follow-up period of 4 years. The suprachoroidal delivery approach, placed proximate to the choroid, offers the distinct advantage of facilitating the entry of growth factors (GFs) into the choroidal bloodstream, thereby effectively managing the steady secretion of GFs to the choroidal and retinal structures.

This pioneering approach has been conclusively shown to be safe, with no associated complications. Given the advanced disease stage of the participants enrolled in our study, particularly considering their age and baseline BCVA, it is likely that the interaction between GFs and their receptors was suboptimal. As a result, the observed outcomes may not fully reflect the potential therapeutic response that could be achieved in patients at earlier or moderate stages of the disease. Nevertheless, despite the heterogeneity of the study population, our findings are encouraging, demonstrating an absence of serious adverse events and offering promise for future investigations involving patients at earlier phases of disease progression.

Despite these promising findings, long-term studies are needed to determine whether suprachoroidal implantation of MSCs leads to their integration into other ocular structures or if donor cells persist within the host eye. Specifically, it is crucial to assess whether some stem cells bypass the blood-retinal barrier and migrate into non-target tissues, such as the retina or choroid.

To visualize donor cells after transplantation, various techniques are available, depending on the type of stem cells and their labeling. OCT is effective for monitoring structural changes in the retina and choroid post-transplantation. However, direct visualization of individual cells is not possible without contrast labeling, which is not feasible in clinical studies.

In preclinical research, stem cells can be labeled with dyes such as PKH26 or green fluorescent protein, allowing tracking through bioluminescence. However, these methods are primarily used in animal studies, as fluorescence diminishes over time.

Given these limitations, the authors relied exclusively on OCT imaging to evaluate retinal and choroidal changes following transplantation.

### Limitations

These factors represent the primary limitations of our study.

1. Genetic analysis and its correlation with functional test outcomes were not incorporated into this investigation. Genetic profiling could provide valuable insights into the differential responses to stem cell therapy among various genetic subtypes of RP. As genetic diagnostics become increasingly integral to clinical practice, it will likely become possible to better delineate the impact of MSC administration for different genetic categories of RP. Consequently, despite the heterogeneity of the study cohort and the absence of molecular diagnostic data, our findings suggest that suprachoroidal MSCs are capable of sustaining retinal neuroenhancement.
2. The current diagnostic and follow-up tools—primarily based on BCVA changes, scotoma detection via VF testing, ocular electrophysiology, and OCT imaging. Although visual field (VF) testing has been demonstrated to be a sensitive method for quantifying preserved central visual function, it is associated with certain limitations. VF assessment is a behavioral and subjective technique that depends heavily on patient cooperation and familiarity with the testing procedure. In contrast, more objective modalities such as microperimetry may provide a more reliable and reproducible evaluation of central VF.
3. To enhance the sensitivity of our assessments, more advanced functional tests are needed. FST testing represents one such promising modality. Unfortunately, most of our patients do not have serial FST data, as the test was only recently introduced in our clinic. Beyond its diagnostic value in detecting early neuroretinal dysfunction, FST may also prove valuable in monitoring treatment efficacy.
4. This study did not include patients in the early stages of RP. Future investigations comparing outcomes across different stages of the disease will be essential to determine which patient populations are most likely to benefit from stem cell implantation therapies.

## Conclusions

RP is an inherited disease characterized by progressive retinal degeneration, which can ultimately lead to complete blindness. In the natural history cohort without intervention, both functional and anatomical parameters deteriorated significantly over time. Currently, therapeutic strategies for RP include gene therapy, optogenetics, and cell-based therapies. Irrespective of the underlying genetic mutation, clinical experience suggests that cell-mediated therapies are noteworthy due to their potential to enhance retinal electrical responses. Among these approaches, MSC therapy has been shown to significantly decelerate and stabilize disease progression. It is reasonable to hypothesize that greater therapeutic benefits could be achieved if MSC therapy is administered during the earlier stages of the disease, when a substantial number of photoreceptors remain viable. Continued follow-up will provide further insights into the long-term efficacy and safety profile of MSC-based interventions.

## Data Availability

The data underlying this article are available in the article and in its online supplementary material.
